# The Impact of Non-pharmacological Interventions on Blood Pressure Control in Patients With Hypertension: A Systematic Review

**DOI:** 10.7759/cureus.48444

**Published:** 2023-11-07

**Authors:** Omar M Ballut, Abdulrahman A Alzahrani, Raghad A Alzahrani, Aghnar T Alzahrani, Reem A Alzahrani, Mohammad F Alzahrani, Yousef K Alzahrani, Nouf A Alghamdi, Raghad H Alghamdi

**Affiliations:** 1 Internal Medicine, King Fahad Hospital, Al-Baha, SAU; 2 Faculty of Medicine, Al-Baha University, Al-Baha, SAU

**Keywords:** management of hypertension, therapeutic lifestyle modifications, hypertension, blood pressure control, non-pharmacological intervention

## Abstract

Hypertension treatment should involve non-pharmacological interventions such as dietary salt restriction, weight loss, exercise, limiting alcohol intake, and dietary approaches to stop hypertension diet. Significant impacts of these interventions have been suggested for a long time. This systematic review aims to assess the influence of non-pharmacological therapies on hypertension patients’ ability to control their blood pressure. The review will concentrate on randomized controlled trials examining how non-pharmacological therapies affect blood pressure regulation in hypertension patients.

A systematic review was conducted to investigate the impact of non-pharmacological interventions on blood pressure control in patients with hypertension. A comprehensive search for relevant studies was conducted. The following electronic databases were searched: EMBASE, OVID-MEDLINE, and PubMed. The search covered the period between January 2000 and August 2023. The search strategy included a combination of keywords related to hypertension, non-pharmacological interventions, and blood pressure control.

A thorough literature evaluation of papers from the EMBASE, OVID-MEDLINE, and PubMed databases was part of the procedure for choosing the studies. Combinations of the keywords telemedicine, primary care, and effectiveness were used for the search. Only studies published in English between January 2000 and August 2023 were included in the search. Through database searching, 862 entries were found, of which 321 were from EMBASE, 112 from OVID-MEDLINE, and 429 from PubMed. After duplicate records were eliminated, 117 records were checked for eligibility. Of these, 100 were disregarded for a variety of reasons, including not relevant to the objectives of the study (n = 63), abstracts or reviews (n = 8), and studies that failed to present interesting research findings (n = 36). The eligibility of the remaining 10 full-text publications was evaluated. Ten articles passed the inclusion tests and were added to the research after a thorough evaluation.

Lifestyle modifications are important and have a significant impact on controlling hypertension and a positive impact on reducing blood pressure. Combination therapy is more effective; however, adherence to the modifications is the most important factor affecting the outcomes.

## Introduction and background

High blood pressure (BP), or hypertension, is a major global public health issue [1,2]. Hypertension was defined in 2017 by the American College of Cardiology/American Heart Association (ACC/AHA) as a cutoff point of systolic blood pressure (SBP) ≥130 mmHg and/or diastolic blood pressure (DBP) ≥80 mmHg, using an average of ≥2 readings obtained on ≥2 occasions to estimate the individual’s BP levels [3]. The World Health Organization (WHO) estimates that hypertension affects 1.13 billion people worldwide and causes more than 7.5 million fatalities each year [4]. Chronic hypertension can cause serious problems such as stroke, heart attack, kidney damage, and blindness if not managed appropriately [5]. Typically, lifestyle changes, pharmaceutical therapies, or a mix of the two are used to treat hypertension [6]. However, it has been demonstrated that non-pharmacological therapies, such as dietary adjustments, exercise, and stress management, have a considerable impact on BP regulation [7,8].

Numerous risk factors play a role in the pathophysiology of hypertension, which is complicated and multifactorial. Age, family history, obesity, physical inactivity, excessive alcohol consumption, and a diet high in sodium are the main risk factors for hypertension [9]. Owing to its tendency to develop symptoms only when it has advanced to a severe stage, hypertension is frequently referred to as the silent killer [10].

The goal of hypertension treatment is to lower BP to delay or stop the onset of problems. Standard hypertension treatment includes pharmaceutical therapies, such as antihypertensive drugs, as well as lifestyle changes such as dietary adjustments, exercise, and weight loss [11]. Non-pharmacological approaches, however, have drawn more attention recently due to their potential to lower BP levels without the use of drugs [11].

Interventions that do not employ medications are known as non-pharmacological interventions. These interventions frequently center on dietary adjustments, physical activity, stress reduction, and quitting smoking [12]. According to research, non-pharmacological therapies can significantly affect BP control in hypertensive patients.

This systematic review aims to assess the influence of non-pharmacological therapies on hypertension patients’ ability to control their BP. The review will concentrate on randomized controlled trials (RCTs) examining how non-pharmacological therapies affect hypertension patients’ BP regulation.

## Review

Methodology

A systematic review was conducted to investigate the impact of non-pharmacological interventions on BP control in patients with hypertension. The review focused on RCTs that investigated the effects of non-pharmacological interventions on BP control in hypertensive patients.

Search strategy

A comprehensive search for relevant studies was conducted using the following electronic databases: EMBASE, OVID-MEDLINE, and PubMed. The search covered the period between January 2000 and August 2023. The search strategy included a combination of keywords related to hypertension, non-pharmacological interventions, and BP control.

Eligibility criteria

The inclusion criteria for this review were as follows: RCTs that investigated the impact of non-pharmacological interventions on BP control in patients with hypertension, studies that involved adult participants aged 18 years or older with a diagnosis of hypertension, studies that reported BP (SBP and DBP) as an outcome measure, and studies that were published in the English language between January 2000 and August 2023.

The exclusion criteria were as follows: studies that were not RCTs, studies that involved participants who did not have hypertension, studies that did not report BP as an outcome measure, and studies that were published before January 2000.

Data extraction

Data extraction was performed independently by two reviewers using a standardized data extraction form. The following information was extracted from each study: (1) study characteristics (author, year of publication, study design, sample size, duration of follow-up), (2) participant characteristics (age, gender, ethnicity, comorbidities), (3) intervention details (type of non-pharmacological intervention, duration, frequency, intensity), (4) outcome measures (SBP and DBP), and (5) results (mean and standard deviation of BP, effect size, p-value).

Ethics and dissemination

No ethical approval was required for this systematic review as it used published data. The results of this review were disseminated through publication in a peer-reviewed journal and presentation at relevant conferences.

Results

**Figure 1:**
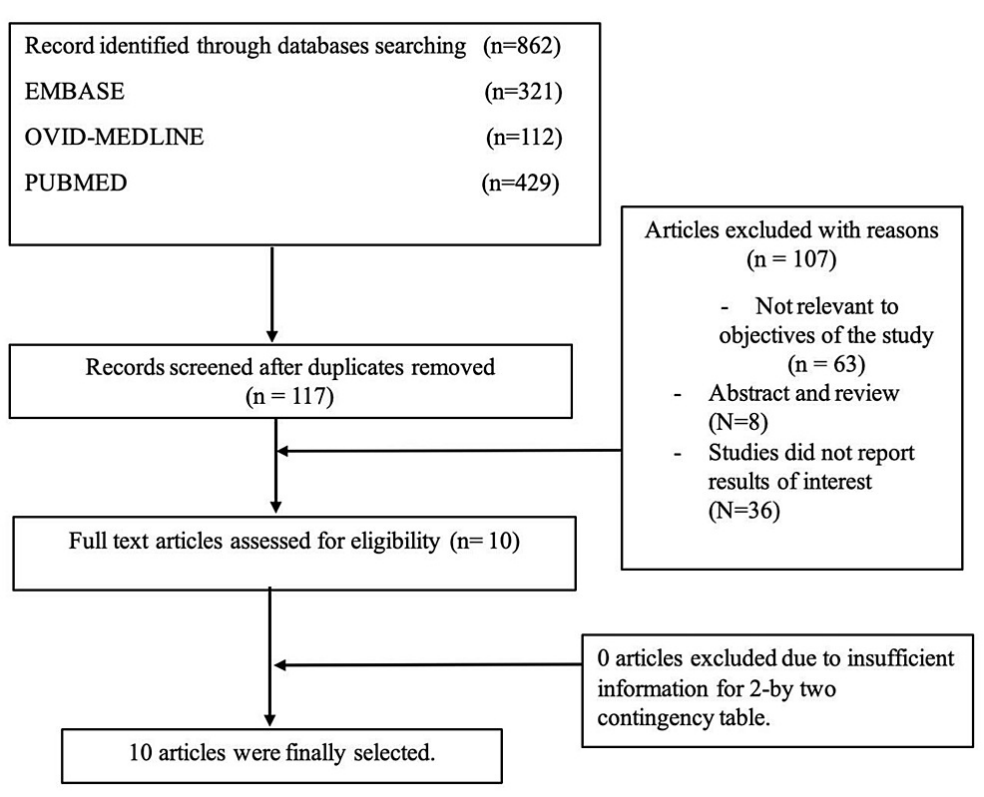
Preferred Reporting Items for Systematic Reviews and Meta-Analyses figure showing the steps of study selection for this systematic review.

A thorough evaluation of papers from the EMBASE, OVID-MEDLINE, and PubMed databases was part of the procedure for choosing the studies. Combinations of the keywords telemedicine, primary care, and effectiveness were used for the search. Only studies published in English between January 2000 and August 2023 were included in the search. Through database searching, 862 entries were found, of which 321 were from EMBASE, 112 from OVID-MEDLINE, and 429 from PubMed. After duplicate records were eliminated, 117 records were checked for eligibility. Of these, 100 were disregarded for various reasons, including not relevant to objectives of the study (n = 63), abstracts or reviews (n = 8), and failing to present interesting research findings (n = 36). The eligibility of the remaining 10 full-text publications was evaluated. Ten articles passed the inclusion tests and were added to the research after a thorough evaluation (Figure 1).

A total of 10 studies were included in this review with a total of 1,462 participants (Table 1). The studies were conducted between 2003 and 2017 in a variety of settings, including community, clinical, laboratory, and university settings. The sample sizes ranged from 17 to 810 participants. Most studies included both male and female participants with ages ranging from 18 to 70 years. Five studies had follow-up periods of six months or less, while four studies had follow-up periods of one year or more. The characteristics of the included studies were similar, with some variation in sample sizes, settings, and follow-up durations. Most studies were RCTs, although some were quasi-experimental. The populations varied from general community samples to sedentary participants to patients with elevated BP or hypertension. However, collectively the studies provided useful information on the effects of exercise on BP in a range of populations.

**Table 1:** Characteristics of included studies. N/A = not available; BP = blood pressure

Study	Year	Design	Setting	Sample size	Gender	Age	Follow-up
Zou et al. [13]	2017	Pilot randomized controlled trial	Community, Canada	60	31 women, 29 men	Mean age: 62.0 years	8 weeks
Sarkkinen et al. [14]	2011	Randomized, double-blind, placebo-controlled study	Finland	45	22 women, 23 men	Mean age: 55 years	8 weeks
Zhao et al. [15]	2014	Randomized controlled trial	Tibetan Autonomous Region, China	282	166 women, 116 men	Mean age: 63.1 years	3 months
Hansen et al. [16]	2012	Randomized controlled trial	Denmark	88	39 women	Mean age: 52.0 years	12 weeks
Badrov et al. [17]	2013	Quasi-experimental	University laboratory	24 hypertensives	11 females	Mean age: 64 years	2 months
Taylor et al. [18]	2003	Randomized controlled trial	University laboratory	17 hypertensives	10 males, 7 females	Mean age: 67.5 years	6 months
Edwards et al. [19]	2011	Randomized controlled trial	University laboratory	52 sedentary participants with elevated BP	25 males, 27 females	18–70	6 months
Stewart et al. [20]	2005	Randomized controlled trial	Community setting	51 exercisers, 53 controls	25 males, 26 females in exercisers; 26 males, 27 females in controls	Mean age: 63.6 years	6 months
Elmer et al. [21]	2006	Randomized controlled trial	Clinical centers	810 adult volunteers with pre-hypertension or stage 1 hypertension	N/A	N/A	18 months
Burke et al. [22]	2005	Randomized controlled trial	Research studies unit	241 overweight hypertensive patients	N/A	N/A	1 year

Ten studies were included in this review with a range of intervention strategies and effects on hypertension (Table 2). Dietary interventions such as the dietary approaches to stop hypertension (DASH)-sodium-CC (DASHNa-CC) [13] and low-sodium, high-potassium salt [15] demonstrated reductions in SBP ranging from 3.8 to 8.2 mmHg. Exercise-based interventions including aerobic interval training [16] and combined aerobic and resistance training [20] showed decreases in SBP from 5.3 to 12 mmHg. Isometric handgrip training also significantly reduced resting and ambulatory BP in several studies [17,18]. Multicomponent behavioral interventions incorporating diet, exercise, and medication changes demonstrated effects on variables associated with hypertension as well as reduced odds of hypertension at follow-up [21,22]. Collectively, the included studies provided evidence that a variety of lifestyle intervention strategies can achieve clinically meaningful reductions in hypertension.

**Table 2:** Intervention strategies and outcomes. DASHNa-CC = dietary approaches to stop hypertension with sodium reduction for Chinese Canadians; BP = blood pressure; SBP = systolic blood pressure; DBP = diastolic blood pressure; BMI = body mass index

Study	Intervention	Numerical decrease/increase in hypertension
Zou et al. [13]	DASHNa-CC intervention	Decrease in SBP: 3.8 mmHg
Sarkkinen E et al. [14]	Smart Salt	Reduction in SBP: -7.5 mmHg
Zhao et al. [15]	Low-sodium and high-potassium salt substitute	Reduction in SBP: -8.2 mmHg; reduction in DBP: -3.4 mmHg
Hansen et al. [16]	Aerobic interval training	Reduction in ambulatory 24-hour BP: SBP -12 mmHg, DBP -8 mmHg
Badrov et al. [17]	Isometric handgrip training (IHGT)	Resting BP: Δ8/5 mmHg; systolic BP reactivity to the SST (Δ7 mmHg) and IHGT (Δ8 mmHg) was reduced
Taylor et al. [18]	Isometric handgrip training	Resting systolic pressure and mean arterial pressure decreased; SBP decreased in the training group (156 ± 9.4 mmHg to 137 ± 7.8 mmHg) versus the control group (152 ± 7.8 mmHg to 144 ± 11.8 mmHg)
Edwards et al. [19]	Exercise-only or Exercise plus DASH diet	Both intervention groups showed increases in heart rate recovery (HRR) and significant reductions in BP from pre- to post-intervention; BP post-intervention was significantly predicted by change in HRR when controlling for pre-BP, age, gender, and BMI
Stewart et al. [20]	Combined aerobic and resistance training	Mean decreases in SBP and DBP, respectively, were 5.3 and 3.7 mmHg among exercisers and 4.5 and 1.5 mmHg among controls
Elmer et al. [21]	Multicomponent behavioral intervention	Both behavioral interventions statistically significantly reduced weight, fat intake, and sodium intake; the odds ratios for hypertension at 18 months were 0.83 (95% CI = 0.67 to 1.04) for the established group and 0.77 (95% CI = 0.62 to 0.97) for the established plus DASH group
Burke et al. [22]	Multifactorial lifestyle modification	Mean 24-hour ambulatory BP changed significantly with the lifestyle program (-4.1/-2.1 ± 0.7/0.5 mmHg) compared to controls (-1.0/-0.3 ± 0.5/0.4 mmHg); 41% in the control group and 43% in the program group maintained the drug withdrawal status

Table 3 presents the factors affecting the outcomes of the included studies. These factors varied from study to study and included adherence to the trial protocol, satisfaction with the intervention, reduction in sodium intake, use of Smart Salt, and duration of intervention. For example, Sarkkinen et al. found that the use of Smart Salt was a significant predictor of a reduction in SBP. Zou et al. found that adherence to the DASHNa-CC intervention was associated with a greater decrease in SBP [13]. Hansen et al. found that higher adherence to the aerobic interval training intervention was associated with a greater reduction in ambulatory 24-hour BP [16]. Edwards et al. found that adherence to the exercise-only intervention was associated with a greater decrease in SBP, while adherence to the exercise plus DASH diet intervention was associated with a greater decrease in DBP [19]. Burke et al. found that higher adherence to the lifestyle program was associated with a greater reduction in BP [22].

**Table 3:** Factors affecting outcomes. BP = blood pressure; SBP = systolic blood pressure; SST = serial subtraction; IHGT = isometric handgrip task; DBP = diastolic blood pressure; BMI = body mass index; ABP = arterial blood pressure; HRR = heart rate recovery

Study	Factors affecting outcomes
Zou et al. [13]	Adherence to the trial protocol, satisfaction with the intervention
Sarkkinen et al. [14]	Reduction in sodium intake, use of Smart Salt
Zhao et al. [15]	Use of low-sodium and high-potassium salt substitute, length of the intervention
Hansen et al. [16]	Type and intensity of exercise, duration of the intervention
Badrov et al. [17]	Pretraining SBP reactivity to the SST and IHGT was correlated with the decrease in SBP post-IHG training
Edwards et al. [19]	Change in HRR significantly predicted BP post-intervention when controlling for pre-BP, age, gender, and BMI
Stewart et al. [20]	Body composition improvements explained 8% of the SBP reduction and 17% of the DBP reduction
Elmer et al. [21]	Both behavioral interventions statistically significantly reduced weight, fat intake, and sodium intake; the odds ratios for hypertension at 18 months were 0.83 (95% CI = 0.67 to 1.04) for the established group and 0.77 (95% CI = 0.62 to 0.97) for the established plus DASH group
Burke et al. [22]	The lifestyle program resulted in a greater decrease in mean 24-hour ABP compared to controls, and 41% in the control group and 43% in the program group maintained the drug withdrawal status; the intervention was more effective among those who were not on medication at baseline, had higher BMI, higher baseline ABP, or were more adherent to the program

Discussion

The available research on how lifestyle changes affect hypertension was summarized in this study. The included research showed that a range of dietary, exercise, and behavioral therapies can reduce BP and the risk of hypertension in a clinically significant manner. However, the level of effectiveness varies depending on elements such as adherence to the intervention protocol and length of the intervention.

The suggested intervention’s adherence has a significant impact on the results. According to several studies [13,16,18], higher adherence to the intervention was linked to larger drops in BP. This confirms previous studies which showed that adherence to medications or lifestyle interventions has a beneficial impact on BP [23-25]. This implies that patients must adhere to the suggested dietary adjustments, exercise regimens, or medication changes to achieve the best results [25]. Ineffective attempts to change one’s lifestyle may not significantly lower BP [26]. Results may be improved by encouraging patients to follow interventions with the help of education, counseling, and follow-up. A patient-centered approach needs to be stressed to encourage adherence to BP therapy [27]. When patients and providers collaborate to develop a treatment plan and set goals, patients feel more empowered and committed. Techniques used in motivational interviewing may reveal ambivalence and obstacles that can then be cooperatively resolved. For sustained adherence over time, follow-up and accountability via check-ins and remote monitoring are also crucial [27,28]. New healthy habits are strengthened through social support from family, friends, and support groups [26]. The most promising strategies for good adherence and eventual BP reduction are those that are specifically customized to the needs, preferences, and circumstances of individual patients.

The length of the lifestyle intervention also has an impact on the results, with longer interventions typically leading to bigger BP decreases. SBP reductions ranged from 4.5 to 12 mmHg in trials with follow-up lasting six months or more [16,19-22]. Studies with shorter follow-up durations, however, showed reductions from 3.8 to 8.2 mmHg [13-15]. This suggests that for lifestyle changes to fully affect hypertension, consistent effort over months or years may be necessary [29,30]. There might be a need for support services and longer-term behavioral therapies.

While the majority of research only examined single modalities such as diet or exercise, multicomponent therapies that combined dietary, physical activity, and pharmaceutical changes demonstrated some of the most positive outcomes [19,21,22]. This shows that to achieve the best results, particularly for patients with more severe hypertension, a multifaceted strategy targeting numerous risk factors may be required. Multimodal therapies might sometimes be more complicated and difficult for patients [6]. If the treatment plan becomes overly detailed, adherence may decline. Therefore, even though combination interventions have a greater chance of being beneficial, they should be kept as simple as possible and customized to the unique requirements and skills of each patient. Maintaining this balance can be made easier with regular evaluation and the ability to modify the plan as needed. Multimodal therapies may significantly lower BP without placing an undue strain on patients if they are patient-centered.

This study shows that altering one’s lifestyle has the potential to reduce BP and treat hypertension. However, there are still some important restrictions. Numerous studies lacked generalizability, had small sample sizes, and shorter follow-up durations. Future research should use larger, more representative samples, and run for longer periods of time. The quality of the evidence would also be improved by adherence monitoring and standardized intervention regimens.

## Conclusions

While lifestyle changes have the potential to reduce the risk of developing hypertension, additional study is required to identify the most effective intervention options and how to best encourage long-term patient adherence. Diet and exercise regimens may be used as a first-line or supplemental treatment for many people with increased BP with the right treatments that are customized to each patient’s needs.
